# Why COVID-19 Transmission Is More Efficient and Aggressive Than Viral Transmission in Previous Coronavirus Epidemics?

**DOI:** 10.3390/biom10091312

**Published:** 2020-09-11

**Authors:** Fatma Elrashdy, Elrashdy M. Redwan, Vladimir N. Uversky

**Affiliations:** 1 Department of Endemic Medicine and Hepatogastroenterology, Kasr Alainy School of Medicine, Cairo University, Cairo 11562, Egypt; fatmaelrashdy@kasralainy.edu.eg; 2 Biological Science Department, Faculty of Science, King Abdulaziz University, P.O. Box 80203, Jeddah 21589, Saudi Arabia; 3 Department of Molecular Medicine and USF Health Byrd Alzheimer’s Research Institute, Morsani College of Medicine, University of South Florida, Tampa, FL 33612, USA; 4 Institute for Biological Instrumentation of the Russian Academy of Sciences, Federal Research Center “Pushchino Scientific Center for Biological Research of the Russian Academy of Sciences”, Pushchino, 142290 Moscow, Russia

**Keywords:** severe acute respiratory syndrome coronavirus 2, SARS-CoV-2, coronavirus disease 2019, COVID-19, viral infection, virus-host interaction

## Abstract

Severe acute respiratory syndrome coronavirus 2 (SARS-CoV-2) is causing a pandemic of coronavirus disease 2019 (COVID-19). The worldwide transmission of COVID-19 from human to human is spreading like wildfire, affecting almost every country in the world. In the past 100 years, the globe did not face a microbial pandemic similar in scale to COVID-19. Taken together, both previous outbreaks of other members of the coronavirus family (severe acute respiratory syndrome (SARS-CoV) and middle east respiratory syndrome (MERS-CoV)) did not produce even 1% of the global harm already inflicted by COVID-19. There are also four other CoVs capable of infecting humans (HCoVs), which circulate continuously in the human population, but their phenotypes are generally mild, and these HCoVs received relatively little attention. These dramatic differences between infection with HCoVs, SARS-CoV, MERS-CoV, and SARS-CoV-2 raise many questions, such as: Why is COVID-19 transmitted so quickly? Is it due to some specific features of the viral structure? Are there some specific human (host) factors? Are there some environmental factors? The aim of this review is to collect and concisely summarize the possible and logical answers to these questions.

## 1. Introduction

In addition to the seasonal flu that annually infects 9% of the world population and causes 291,000–600,000 deaths each year (death rate around 0.1%), the past 100 years witnessed several outbreaks of viral infections, such as the 1918 influenza pandemic (500 million infected; 50 million died; mortality rate 10%), 2002–2004 severe acute respiratory syndrome (SARS) outbreak (8098 cases; 774 deaths; mortality rate 9.5%), 2009–2010 H1N1 influenza pandemic (1.649 billion infected; i.e., 24% of the global population (~61 million cases in the USA); 284,000 died (~12,500 deaths in the USA); mortality rate 0.02%), 2012–2020 middle east respiratory syndrome (MERS) outbreak (2519 cases; 866 deaths; mortality rate 34.4%), 2014–2016 Ebola outbreak (~28,650 cases across 10 countries; 11,325 deaths; mortality rate 39.5%), and currently developing coronavirus disease 2019 (COVID-19) pandemic. It is difficult to make a projection of the final outcomes of the COVID-19 pandemic, which is still developing, but the currently available data are staggering (as of 4 September 2020): there are almost 26.8 million COVID-19 cases in 213 countries and territories around the world and two international conveyances, with more than 877,000 patients died. Although current statistics indicate that 3.3% of the SARS-CoV-2 infected have died worldwide, the COVID-19 mortality rates are not equal in all affected territories and vary in a wide range in different countries (from 0.56% in Iceland to >18% in France). Of these six global outbreaks of viral infections, three were caused by coronaviruses (SARS, MERS, and COVID-19), of which COVID-19 is characterized by the most efficient and aggressive transmission. In fact, COVID-19, which is caused by the infection with severe acute respiratory syndrome coronavirus 2 (SARS-CoV-2, also known as 2019 new CoV, 2019-nCoV), is spreading like wildfire worldwide, affecting almost every country in the world. Taken together, both previous outbreaks of other members of the coronavirus family (SARS-CoV and MERS-CoV) did not produce even 1% of the global harm already inflicted by COVID-19. Furthermore, in addition to SARS-CoV, MERS-CoV, and SARS-CoV-2 (all are β-CoVs of the B and C lineage), there are four other coronaviruses (CoVs) capable of infecting humans (HCoVs), which circulate continuously in the human population. These are HCoV-OC43 [1,2] and HCoV-HKU1 [3] (β-CoVs of the A lineage or β1CoVs), and HCoV-229E [4,5] and HCoV-NL63 [6,7] (α-CoVs). Being identified in the late 1960s (HCoV-229E and the HCoV-OC43) [8,9,10,11,12] and in 2004-2005 (HCoV-NL63 [6,7,13] and HCoV-HKU1 [3]), these HCoVs are known to be responsible for 3–10% cases of the common cold and short-term upper respiratory infections that occur mainly in winter, with a short incubation time [14,15], with about 2% of the human population being healthy carriers of an HCoV [16,17]. Although these HCoV strains can also cause more serious diseases of the lower respiratory tract, such as bronchitis, bronchiolitis, and pneumonia, especially in newborns or infants, elderly people, and immunocompromised patients [16,17], their phenotypes are generally mild, and as a result, these four HCoVs received relatively little attention.

These dramatic differences between infection with HCoVs, SARS-CoV, MERS-CoV, and SARS-CoV-2 raise many questions, such as: Why is COVID-19 transmitted so quickly? Is it due to some specific features of the viral structure? Are there some specific human (host) factors? Are there some environmental factors? The aim of this study is to collect and concisely summarize the possible and logical answers to these questions.

## 2. Intrinsic Viral Factors

CoVs belong to the subfamily *Coronavirinae* of the *Coronaviridae* family (which also includes the *Torovirinae* subfamily) in the order *Nidovirales*. They are divided into four genera, namely α-, β-, γ-, and δ-CoVs, with β-CoVs being further separated into A, B, C, and D lineages or clades [18]. Of four CoV genera, α- and β-CoV are able to infect mammals (including humans and domestic animals), while γ- and δ-CoV tend to infect birds. The emergence of human-infecting CoVs is likely associated with cross-species transmission events [19]. For example, SARS-CoV-2 shows close genetic similarity to bat coronaviruses [20,21,22,23]. SARS-CoV and MERS-CoV are zoonotic viruses that crossed the species barrier using bats/palm civets [24] and dromedary camels [25], respectively. Similarly, HCoV-OC43 originated from a zoonotic transmission event of a bovine coronavirus (BCoV) [26,27], HCoV-HKU1 from a bat coronavirus [28], and HCoV-NL63 originated from ARCoV.2 (Appalachian Ridge CoV) detected in North American tricolored bat (*Perimyotis subflavus*) [29]. Finally, HCoV-229E originated in hipposiderid bats, with camelids serving as potential intermediate hosts [30].

The single-stranded RNA genome of SARS-CoV-2 includes 29,903 nucleotides and encodes three structural proteins, such as spike glycoprotein (S), an envelope protein (E), membrane protein (M), and nucleocapsid protein (N), six accessory proteins, encoded by *ORF3a*, *ORF6*, *ORF7a*, *ORF7b*, and *ORF8* genes, and several non-structural proteins (NSPs) in the form of a polyprotein encoded by a large, 5′-located *ORF1ab* replicase gene that covers more than two-thirds of the viral genome [31,32,33]. This *ORF1ab* replicase gene encodes a set of NSPs that play a number of important roles in viral replication. This replicase gene encodes the overlapping polyproteins named pp1a and pp1ab, which are necessary for viral replication and transcription. The longer pp1ab is a 7073 amino acid-long polypeptide containing 15 non-structural proteins. NSP1, NSP2, and NSP3 are released from polyprotein via proteolytic processing using a viral papain-like proteinase (NSP3/PL^Pro^), whereas the rest of NSPs are cleaved by another viral 3C-like proteinase—NSP5/3CL^Pro^ or main protease M^pro^—that utilizes 11 or more conserved sites to digest the polyprotein. This digestion starts with an autocatalytic cleavage of this enzyme itself from pp1a and pp1ab.

Based on the evaluation of the levels of intrinsic disorder in the nucleocapsid (N) and membrane (M) proteins of SARS-CoV-2, it was proposed that this virus is characterized by high resilience to the conditions outside the body and in body fluids, suggesting that SARS-CoV-2 belongs to viruses with intermediate levels of both respiratory and fecal-oral transmission potentials [34,35], which favor alternative ways for the COVID-19 transmission vertically and horizontally.

An important feature that differentiates β-CoVs of the B and C lineages (SARS-CoV, MERS-CoV, and SARS-CoV-2) from β-CoVs of the A lineage (β1CoVs) is the lack of hemagglutinin-esterase (HE) protein, which is present in toroviruses, influenza C and D viruses, and in β1CoVs [36,37,38,39,40]. HE is a receptor-binding/receptor-destroying viral protein interacting with the 9-*O*-acetylated sialic acids (9-*O*-Ac-Sias) [38], which are the glycan components commonly present in mammals and birds [41]. Therefore, in β1CoVs, both spike and HE proteins bind 9-*O*-Ac-Sias, whereas virus elution is promoted by receptor destruction via the action of the HE esterase domain. These opposing activities of receptor binding and receptor destruction define dynamic and reversible attachment of β1CoV to sialoglycans. The sialate-*O*-acetyl-esterase activity promotes escape from attachment to non-permissive host cells or decoy and facilitates the release of viral progeny from infected cells [42]. Curiously, it was shown that the HE lectin function is progressively lost during the in-host evolution of the human β1CoVs, HCoV-OC43, and HCoV-HKU1 [43]. Spike proteins of MERS-CoV interact with a specific receptor, dipeptidyl peptidase-4 (DPP4), which is a key factor in the signaling and activation of the acquired and innate immune responses in infected patients [44]. On the other hand, the host cell entry of SARS-CoV, HCoV-NL63, and SARS-CoV-2 is mediated by interaction with the angiotensin-converting enzyme-2 (ACE2) receptors, which are expressed in the brain, gut, heart, kidney, lung (particularly in type 2 pneumocytes and macrophages), vessels, and testis [45]. However, besides this protein membrane receptor, the host cell entry of HCoVs, including SARS-CoV-2, also depends on the sialic-acid-containing glycoproteins and gangliosides, which might act as primary attachment factors for viruses along the respiratory tract [38]. In fact, the N-terminal domain (NTD) of the spike (S) glycoprotein of SARS-CoV-2 was shown to contain a ganglioside-binding site that can be efficiently blocked by chloroquine (CLQ) and its more active derivative, hydroxychloroquine (CLQ-OH) [46]. Therefore, the SARS-CoV-2 S protein acts on both protein and 9-*O*-acetylated sialic acid-containing receptors, with the receptor-bind domain (RBD) being involved in ACE2 receptor recognition, and the NTD being responsible for finding a ganglioside-rich landing area (lipid raft) at the cell surface [46]. It was hypothesized that the interaction of S protein with the lipid rafts defines an adequate positioning of the viral S protein at the first step of the infection process [46]. Importantly, the evolutionary analysis revealed that the ganglioside-binding subdomain (residues 111–162) of the NTD is completely conserved in 11 clinical isolates of SARS-CoV-2 of various geographic origins. Furthermore, this subdomain is also completely conserved in the bat coronavirus RaTG13, but noticeable variability is detected in other bat SARS-like and human SARS-CoVs, suggesting that higher levels of SARS-CoV-2 contagiousness in comparison with previously characterized HCoVs can be attributed to recent evolution [46].

Just a few weeks after the first reports on COVID-19 infection, it was revealed that the virus enters the lung alveolar type II (AT2) via the ACE2, which is expressed on the surfaces of the heart, kidneys, intestine, and lung alveolar epithelial cells. Here, a specific role is played by the spike glycoprotein S. In fact, the S glycoproteins of coronaviruses have two subunits—S1 and S2. The S1 subunit binds to the ACE2 enzyme, via its receptor-binding domain (RBD), on the cell membrane [47,48], and S2 fuses with the cell membrane [49]. Although the genome of SARS-CoV-2 shares 79.6% sequence identity to SARS-CoV, and although SARS-CoV-2 is capable of using the same cell entry receptor (ACE2) as SARS-CoV to infect humans [21,50], the affinity of SARS-CoV-2 spike protein to the human ACE2 is ~10–20 fold higher than that of the SARS-CoV spike protein [51,52]. This is because of the presence of the distinctive structural differences between the receptor-binding domains (RBDs) of the spike proteins from SARS-CoV and SARS-CoV-2, which represent energetically favorable changes in the amino acid sequence for the more efficient interaction of the SARS-CoV-2 spike protein with the ACE2 receptor. In fact, the local environment within the ACE2 receptor allows SARS-CoV-2-specific residues in the RBD of the spike protein to make a significant number of electrostatic stabilizing interactions. Furthermore, the presence of the two capping loops in the RBD of the SARS-CoV-2 spike protein is likely to produce a higher stabilization effect over the interaction with the cellular receptor. These two loops around the RBD of SARS-CoV-2 might promote interaction with the ACE2 receptor, improving the binding to the ACE2 by increasing the number of groups involved. Therefore, these amino acid substitutions and the longer capping loops could explain the increase in the binding affinities in SARS-CoV-2 compared to SARS-CoV. These higher values of affinity might be related to the higher dynamics of the infection and the rapid spread observed for this virus [53]. This is in line with the outputs of the computational analysis showing that when all the residues favoring interaction of the CoV S protein with human ACE2 would be combined into one RBD, this RBD would bind to ACE2 with super affinity, and the corresponding spike protein would mediate viral entry into human cells with super efficiency [54].

Furthermore, SARS-CoV-2 uses the transmembrane protease serine 2 (TMPRSS2, also known as serine protease 10) for the viral spike glycoprotein priming, a process crucial for the viral entry [55]. In fact, host TMPRSS2 priming of the S glycoprotein causes irreversible conformational changes and activation of the S2 subunit, thereby facilitating the fusion of the virus to the cell membrane. The virus with the processed S protein then enters the cell [56,57]. Importantly, S protein of SARS-CoV-2 contains a polybasic cleavage site (RRAR) at the junction of S1 and S2 [51,52,58,59], which defines the effective cleavage by furin and other proteases and has a role in determining viral infectivity and host range [60]. The presence of this unique furin cleavage site within the SARS-CoV-2 spike protein, which is a novel feature setting this virus apart from SARS-CoV, and the almost ubiquitous expression of furin-like proteases could participate in expanding cell and tissue tropism of SARS-CoV-2 and increasing transmissibility and/or altering pathogenicity of this virus [51,52,58,59].

While S2 facilitated the fusion step after proteolysis by TMPRSS2 and furin proteases in a sequential pattern [51,52], there is also evidence suggesting that these enzymes are not the exclusive players in priming S protein for the efficient COVID-19 entry. It is known that airway and alveolar type I and II epithelial cells are expressing other proteases, such as trypsin, kallikrein, and plasminogen, which are also expressed in endothelial cells and which might contribute to the priming of S glycoprotein. The possibility for non-furin proteases to cleave viral envelope proteins is supported by the evidence that the plasmin cleaves the S proteins of SARS-CoV in vitro [61]. Furthermore, S protein of HCoV-HKU1 is cleaved by kallikrein within the S1/S2 region and mediates the entry of HCoV-HKU1 to non-permissive rhabdomyosarcoma cells [62]. Altogether, the S protein of coronaviruses may be cleaved by plasmin, trypsin, cathepsins, elastase, and TMPRSS family members, with such cleavage of S protein mediating the enhancement of the virus entry into the bronchial epithelial cells [61].

One should keep in mind, though, that since the currently available information on the roles of plasmin and other non-furin proteases in cleavage of SARS-CoV in vivo is rather limited, the clinical relevance of such non-furin cleavage is not strictly established. Furthermore, the capability of plasmin to cleave the SARS-CoV-2 envelope proteins remains to be demonstrated [63]. Meanwhile, there is evidence indicating the presence of at least some interplay between SARS-CoV-2 and plasmin. In fact, the enhanced plasmin(ogen) levels and resulting alterations in the fibrin D-dimer levels are the common features observed in the COVID-19 patients [64]. Plasmin proteolytically breaks down excess fibrin and elevates levels of D-dimer (which is a cross-linked dimer of the two smallest fibrin degradation products, with increased D-dimer levels indicating increased fibrinolysis or inability to clear the products from the circulation, and with D-dimer assays being commonly used in clinical practice [65]) and other fibrin degradation products in both bronchoalveolar lavage fluid and plasma, which decreases platelets and results in hemorrhage [64]. Clinical data showed that in the COVID-19 patients, the lungs are the most injured organs, followed by the moderate injury in the heart, liver, kidney, and brain. Systemic microthrombi in the circulatory system and hemorrhage in the affected organs result from the miscoordinated responses between the coagulation and fibrinolysis systems [64]. Coagulation and hemorrhage rank among the top three leading causes of COVID-19-associated death [58].

In addition, elevated levels of plasmin can be related to some other pathological conditions. For example, this protease is known to cleave the subunits of the epithelial sodium channel (ENaC, which is also known as the amiloride-sensitive sodium channel) [64]. ENaC is a heterotrimer of three homologous subunits α or δ, β, and γ, which can be found at the apical membranes of epithelial cells of many tight epithelia of the airway, kidney, and lung. Such plasmin-induced cleavage of the ENaC subunits promotes the flow of Na^+^ ions into the epithelial cells, leading to the dehydration of the air-facing surface of the lungs and alveoli, which is normally lined by a thin film of liquid, and hypertension [64]. Plasmin is a potent protease that cleaves the human γ ENaC subunit at 16 sites, including the cleavage sites of trypsin, chymotrypsin, prostasin, and elastases [66]. Significant harm is induced by the uncontrolled proteolysis of these proteins, which are highly expressed on epithelial cells, considered as the major pathways for Na^+^ entry, and play important roles in maintaining the proper depth of airway and alveolar lining fluids, the reabsorption of edema fluid in injured lungs, and the regulation of salt retention in the collecting tubules [64,67,68,69,70,71,72,73,74]. Of note, the renin-angiotensin system (RAS) is mainly known to regulate blood pressure and Na^+^ reabsorption via its roles in maintaining blood pressure homeostasis [75] and salt and fluid balance [76].

The role of plasmin in the pathogenesis of other viruses is rather well established. For example, it is known that plasmin cleaves the influenza virus hemagglutinin (HA) proteins to enable fusion with the target host endosome [77,78,79,80,81,82]. Besides, the plasminogen (fibrinolytic zymogen, the precursor of plasmin) has been shown to cleave the influenza HA proteins [82,83,84]. The cleavage of HA from the A/WSN/1933 H1N1 influenza virus governs the virus spread in a plasmin-dependent manner [83]. In addition, the replication of both plasmin-sensitive and plasmin-insensitive influenza A virus strains was shown to be enhanced by the plasmin fragment (mini-plasmin), which is preferentially found in the bronchiole epithelial cells, providing further support to the idea that plasmin has several crucial roles in the spread and pathogenicity of the influenza virus [80].

Furthermore, there is a place for other non-furin proteases in viral pathogenesis too. For example, HA proteins from the H1, H2, and H3 subtypes of the influenza virus are sensitive to kallikreins cleavage and can be activated by this protease [85]. Similar to CoVs and influenza viruses, plasmin, trypsin, thrombin, and furin were shown to enhance cytopathology induced by a respiratory syncytial virus (RSV) [86]. Curiously, the cleavage of a target protein by different proteases may enhance or decrease its activities. For example, prostasin (which is a serine protease with trypsin-like substrate specificity that is found in the prostate gland, kidney, bronchi, colon, liver, lung, pancreas, and salivary glands) increases the activity (60–80%) of human ENaC, whereas TMPRSS2 markedly decreases ENaC function and protein levels [87]. Similarly, plasmin is capable of cleaving the subunit of human ENaC at the furin sites [64,88], which may increase the patient complications and subsequently promote viral vertical (and maybe horizontal) tissue tropism and transmissibility [64,89].

TMPRSS2, TMPRSS4, TMPRSS11A, and HAT (human airway tryptase) belong to the type II transmembrane serine proteases (TTSP) family, which includes 19 members, and most of them are expressed in the human respiratory tract [90]. These TTSP can cleave and activate influenza A virus hemagglutinin as well as S proteins of CoVs for host cell entry [91,92]. A comprehensive study detected extensive coexpression of ACE2, TMPRSS2, and HAT in the epithelia of the aerodigestive tract, although exceptions were noted, including the epithelia of the trachea, vocal folds, and epiglottis [92]. Therefore, TMPRSS2 and HAT are present in major viral target cells and could promote viral spread in infected humans [93]. Both enzymes were shown to cleave and activate the HCoV-229E S-protein for cathepsin L-independent virus-cell fusion [93]. Furthermore, TMPRSS2 and HAT were shown to activate all influenza virus subtypes previously pandemic in humans [94,95], and TMPRSS4 was found to activate the HA protein of the 1918 influenza virus [96].

These observations on the roles of various non-furin proteases in the pathogenesis of different viruses raise important questions, such as: Can the plasmin increase the pathogenicity of COVID-19 by cleaving the SARS-CoV-2 S glycoprotein extracellularly, and thereby modulating the ability of this protein to interact with ACE2 receptors of host cells and probably facilitating virus entry and fusion? Can the elevated plasmin(ogen) levels in patients with some pre-existing conditions be considered as one of the avenues for the enhanced susceptibility to SARS-CoV-2 infection and fatality?

There are also some other players from the host protease realm that can contribute to the COVID-19 pathogenesis. In fact, an additional layer of complexity has been added to the interplay between the CoV S protein and host proteases by the observations that not only S of SARS-CoV-2 but also its receptor, ACE2, is proteolytically processed. ACE2 is known to be shed into the extracellular space upon cleavage by the sheddase ADAM17/TACE (disintegrin and metalloproteinase domain-containing protein 17 or tumor necrosis factor (TNF)-alpha-converting enzyme) [93,97,98]. ADAM17 is a 610-residue-long protein that was initially described in 1997 by Black et al. to specifically cleave the precursor of the tumor necrosis factor-α (pro-TNF-α) [99,100]. ACE2 shedding by ADAM17 was first described by Lambert et al., when they studied human HEK293 cells (embryonic kidney cells) expressing human ACE2 (HEK-ACE2) in 2005 [98,99]. In 2008, Haga et al. demonstrated that binding of S protein from SARS-CoV also induced ACE2 shedding by ADAM17 and provided evidence that the ACE2 shedding is important for the uptake of SARS-CoV into the target cells [101]. The up-regulation of ACE2 shedding by ADM17 may inhibit the infectivity of the SARS-CoV [98,99]. Furthermore, it was demonstrated that an ADAM17 inhibitor displays modest antiviral activity in SARS-CoV-infected mice [102]. Furthermore, it was found that TMPRSS2 competes with the metalloprotease ADAM17 for ACE2 processing, but the only cleavage by TMPRSS2 resulted in the augmented SARS-S-driven entry [93].

Since the ACE2 expression levels within the main COVID-19 target, lungs, is relatively low, some researchers suggested that there could be some co-receptors needed for the SARS-CoV-2 entry [103]. Using single-cell RNA sequencing of 13 human tissues, it was established that *ANPEP* (alanyl aminopeptidase), *ENPEP* (glutamyl aminopeptidase), and *DPP4* (dipeptidyl peptidase-4) are the top three genes correlated with *ACE2* [103]. It is known that both ANPEP (which is a membrane-bound broad specificity aminopeptidase) and DPP4 (which is a cell surface glycoprotein receptor) can serve as receptors for HCoVs [104], whereas the involvement of the membrane-bound peptidase ENPEP in virus infection is unclear [103]. One should also keep in mind that human coronaviruses regularly use peptidases as their receptors [48]. ANPEP is the targeted receptor for many viruses belonging to the *Coronaviridae* family, such as porcine epidemic diarrhea virus, HCoV-229E, feline coronavirus, canine coronavirus, transmissible gastroenteritis virus, and infectious bronchitis virus. It is mainly expressing in the colon, ileum, rectum, kidney, liver, and skin [103], demonstrating that the receptor of coronavirus may have similar expression profiles in the human body. Are these data consistent with the fact that CoVs infect similar types of cells and CoV-infected patients share similar clinical symptoms [103]?

Some reports discussed the non-peptidase SARS-CoV receptors as potential avenues for the COVID-19 entry to the host cells. Among such SARS-CoV receptors are DC-SIGN1 (dendritic cell-specific intercellular adhesion molecule (ICAM)-3-grabbing non-integrin 1), CLEC4G (C-type lectin domain family 4 member G), and CLEC4M (C-type lectin domain family 4 member M) [103,105,106]. Furthermore, SARS-CoV-2 may also use integrins as cell receptors via binding to them through a conserved RGD motif (Arg-Gly-Asp, residues 403–405) that is exclusively present in SARS-CoV-2, being absent from other coronaviruses [107]. Curiously, the RGD motif is used by various human non-CoV viruses to interact with their receptors, proteins from the integrin family [108]. Among such human viruses utilizing RGD motifs in their binding to integrins are human adenovirus type 2/5 [109], coxsackievirus A9 [110], human metapneumovirus (HMPV) [111,112], Epstein–Barr virus (EBV, also known as human herpesvirus type 4 (HHV-4)) [113], human cytomegalovirus (HCMV, also known as human herpesvirus type 5 (HHV-5)) [114], Kaposi’s sarcoma-associated virus (HHV-8) [108], and rotavirus (RV) [115].

It is known that the RNA enveloped viruses are using extracellular vesicles (exosomes) to translocate into new host cells [116,117,118]. These vesicles enable the viruses to infect cells in both receptor-dependent and receptor-independent manner and promote viral persistence. They modulate the host immune response, transport populations of viral particles and genomes, increase multiplicities of the ways of viral infection, facilitate cooperative interactions, and enhance the viral replicative fitness [116]. Is SARS-CoV-2 (which is an enveloped RNA virus) follow this pathway to cellular entry and to propagate very quickly? If so, is it dependent or independent on receptor entry? Are there any additional factors that would be increasing the virus entry into the cell? In line with these considerations, we proposed recently that a cellular transport pathway associated with the release of the SARS-CoV-2-loaded exosomes and other extracellular vesicles might represent potential mechanisms for the relapse of the COVID-19 infection [119]. Utilization of such a “Trojan horse” strategy provides SARS-CoV-2 with means to hide viral material within such exosomes or extracellular vesicles during the “silence” time, followed by the re-appearance of the viral RNA in the recovered and discharged COVID-19 patients [119].

In the search for the additional receptor for SARS-CoV cellular entry, SARS pseudovirus or HCoV-NL63 [120,121] were used to explore the possibility of additional routes of the viral entry. It was found that the SARS virus used both S spike and membrane (M) proteins for interaction with common cellular receptors, heparan sulfate proteoglycans (HSPGs), which are present on most cells [122]. These results demonstrated that HSPGs could serve as adhesion receptors that provide the binding sites for SARS-CoV invasion at the early attachment phase. HSPG blockage results in the failure of SARS virus entry even in the presence of the internalization factor ACE2 [123]. From this perspective, it is important to note that lactoferrin/lactotransferrin (LTF) is known to co-localize with the widely distributed cell-surface HSPGs [124,125,126]. SARS-CoV infection activates a host immune response against the virus, where an essential role in the inhibition of the viral infection is played by the innate immune response. In fact, the infection causes up-regulation of several innate immune response-related genes, such as *LFT*, *S100A9*, and *LCN2*, with their corresponding proteins (lactoferrin, S100A9, and lipocalin 2) being involved in the SARS-CoV clearance. As an example, in comparison with the healthy controls, the SARS patients typically showed a 150-fold increase in the *LTF* expression [127]. This is an important observation since lactoferrin is known for its broad virucidal activity, being able to play a role in the suppression of a wide variety of RNA and DNA viruses, such as cytomegalovirus, echovirus, herpes simplex virus, hepatitis C virus, human immunodeficiency virus, human papillomavirus, human polyomavirus, rotavirus, Semliki forest virus, and Sindbis virus [124,125,126]. The entrance of these different viruses into the host cells depends on interaction with common receptors located on the surface of the cells. Among these common receptors that provide the first anchoring sites on the cell surface and thereby promote primary contacts of the virus with the host cells [122] are HSPGs that are broadly distributed on the host cells [124,125,126]. Since lactoferrin can bind to HSPGs, leading to the efficient inhibition of the internalization of some viruses [128], it was hypothesized that such molecular mechanisms could be responsible for the anti-SARS-CoV effects of this protein [120,121]. Is it possible that the SARS-CoV-2 can use a similar entry pathway and utilize HSPGs as its host cell receptors?

**Figure 1:**
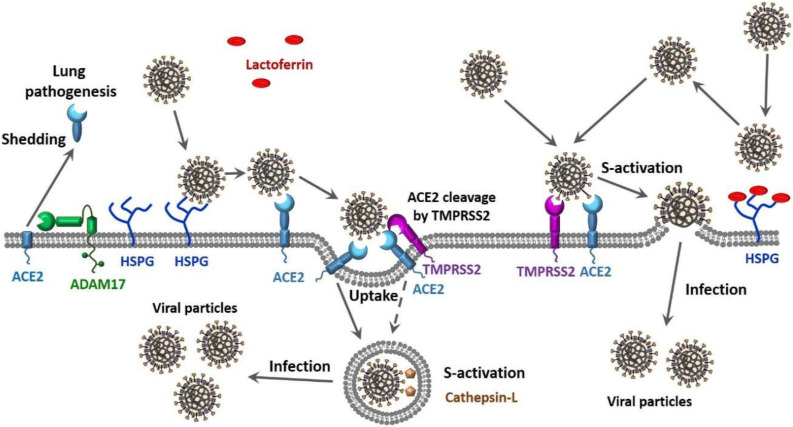
Suggested scenarios for severe acute respiratory syndrome coronavirus 2 (SARS-CoV-2) cellular entry pathways and their potential effects on the viral load and transmission capability.

Finally, there is compelling evidence that CoVs can use multiple pathways to enter the host cell (see Figure 1). In one scenario, the entry of SARS-CoV into cells might occur by direct fusion of envelopes with the plasma membrane at the cell surface [129,130,131]. However, this virus can also take advantage of the endocytic machinery of the target cell. Here, SARS-CoV enters cells by endosomal pathways, where the S protein is activated for fusion by trypsin-like protease in an acidic endosomal environment [130]. The endocytic pathways used by viruses to get into the host cells include macropinocytosis, clathrin-dependent endocytosis, and caveolae-dependent endocytosis, as well as clathrin- and caveolae-independent endocytosis [132,133]. It was pointed out that in the most cases, only one of these pathways is used by a given virus to enter cells, and some viruses might use multiple endocytic pathways to gain entry into host cells [134,135,136,137], with one of these viruses being SARS-CoV [138]. Furthermore, there is also a possibility for the non-endosomal entry of a virus into the host cell. Here, proteases, such as trypsin and thermolysin, promote SARS-CoV cell entry directly from the site where this virus is adsorbed onto the cell surface [139]. Furthermore, protease-mediated SARS-CoV entry from the cell surface was shown to result in a 100- to 1000-fold more efficient infection than entry through endosome [139]. Therefore, SARS-CoV can enter cells via clathrin- and caveolae-independent endocytic pathway or by the non-endosomal pathways that depend on the presence of the proteases [139]. It is known that SARS-CoV-2, which is an enveloped RNA virus, follows this non-endosomal pathway of cellular entry [140].

## 3. Human (Host) Factors

The outcome of SARS-CoV-2 infection is primarily defined by the virus-host interaction, with transmissibility and pathogenicity of SARS-CoV-2 being related to its interplay with host antiviral defense [141]. The first requirements for the successful COVID-19 transmission are the susceptible host with a permissive cell, which carries its receptor. If all these requirements are met, then other factors (such as the receptor orientation, distribution, and structure) will come to play, defining the capabilities of viral particles to be distributed vertically (within the host tissues) and horizontally (within the host population). All this could underhandedly help the virus to be more aggressive (virulent).

Often, the viruses emerging from more resistant hosts have lower overall virulence than viruses emerging from more susceptible hosts. There is correlative evidence supporting the link between the host resistance and virulence evolution [142,143,144]. For example, since virulent strains can be favored over avirulent pathogen strains as a result of the within-host competition, resistant hosts may limit competitive interactions between co-infecting pathogens, thereby hampering the evolution of virulence [145]. The largest adaptive responses in a viral pathogen are achieved via the serial passage of the virus through resistant vs. susceptible hosts, and such adaptive responses are often linked to the most dramatic increases in virulence [146]. It is also possible that the optimal environment for virus adaptation is provided by the hosts with intermediate levels of immunity. This is because such individuals represent an appropriate environment for the optimization of both the pathogen population size and the strength of the immune-mediated selection [147]. All the accumulated data indicate that SARS-CoV-2 may gain some adaptation and enhanced virulence, which globally contributes to its pathogenicity and transmission.

Adaptive T cell immune responses play important roles in the pathogenesis of infectious disease and long-term protective immunity as well as in the development of effective vaccines and therapeutics. The importance of the adaptive T cell immune responses is in the capability of memory T cells induced by the previous pathogens to become activated in the course of new infection with an unrelated heterologous virus, and these memory T cells might be related to the protective immunity and immunopathology [148]. To control the virus, the priming and expansion of the adaptive T cell immune responses are required, and these processes typically take 7–10 days [149]. Viral clearance and capability to reduce the severity of symptoms represent the basis for the T cell-based partial protection against many of acute viral infections, including influenza [150,151,152]. In ten COVID-19 patients placed to an intensive care unit, the presence of SARS-CoV-2-specific cluster of differentiation 4 and 8 (CD4^+^ and CD8^+^) T cells was reported, with the spike surface glycoprotein generating the strongest T-cell responses, and with such SARS-CoV-2-specific T cells predominantly producing Th1 and effector cytokines [153]. The SARS-CoV-2-specific T cells appeared relatively early, and their level increased over time [153]. Curiously, two out of 10 healthy control subjects with no previous exposure to SARS-CoV-2 were shown to also possess low levels of SARS-CoV-2-reactive T cells, suggesting the presence of some cross-reactivity with other human ‘common cold’ causing CoVs [153]. These findings were further validated in an independent study comparing 16 healthy control donors with 42 COVID-19 patients, including 28 mild and 14 severe cases [154]. This study found that in comparison with mild COVID-19 cases, patients with severe cases were characterized by the significantly higher frequency, breadth, and magnitude of memory T cell responses, with the most notable responses being generated by the spike, membrane, and ORF3a proteins [154]. Based on the analysis of the T cell responses against the structural (nucleocapsid protein N) and non-structural (NSP7 and NSP13) proteins of SARS-CoV-2 in 36 individuals recovering from COVID-19, the capability of CD4 and CD8 T cells to recognize multiple regions of the N protein was pointed out [155]. Although there is no information on the duration of the adaptive T cell immune responses against SARS-CoV-2, the recent analysis of the patients recovered from SARS-CoV (i.e., 17 years after the outbreak of SARS in 2003) showed to possess long-lasting memory T cells reactive to the N protein of SARS-CoV [155]. Furthermore, these memory T cells showed vigorous cross-reactivity to the SARS-CoV-2 N protein [155]. Finally, SARS-CoV-2-specific T cells were found in 37 uninfected donors as well. In these individuals with no history of SARS, COVID-19, or known contacts with SARS and/or COVID-19 patients, the SARS-CoV-2-specific T cells possessed a different pattern of immunodominance, being capable of recognition of NSP7 and NSP13 [155].

ACE2 represents the confirmed protein receptor for the SARS-CoV-2 entry into the host cells. The susceptibility of different cohorts of patients to SARS-CoV-2 is correlated with the ACE2 level, and the distribution of target organs that are susceptible to the SARS-CoV-2 infection and the spread of COVID-19-related complications are similar to that of the ACE2 [156]. In fact, entry of the SARS-CoV-2 into the lung alveolar type 2 (AT2) cells is determined by the presence of this receptor. Although ACE2 is reported to be expressed in lung AT2 cells, liver cholangiocyte, colon colonocytes, esophagus keratinocytes, ileal epithelial cells (ECs), rectum ECs, stomach ECs, testis, gallbladder cells, and kidney proximal tubules, its expressing levels are rather low, especially in the lung AT2 cells, where the ACE2 expression levels are 4.7-fold lower than the average expression levels of all ACE2 expressing cell types [103,157]. AT2 cells are considered as alveolar stem cells [158]. They comprise only 5% of the alveoli, but produce the surfactant, a factor essential to maintain lung elasticity, and, most importantly, act as progenitors for AT1 cells, the latter covering 95% of the alveoli and responsible for gas exchange. Therefore, SARS-CoV-2 that targets AT2 cells attacks and kills the lung regenerative pool. Depletion in the AT2 cells and corresponding deficit of surfactants have been previously shown to be associated with the incomplete repair of injured alveolar epithelium and fibrotic obliteration [159]. Therefore, these mechanisms could also explain the development of lung injury in COVID-19 [160]. The low expression of ACE2 in the lung may also suggest the presence of selected cells with up-regulated ACE2 expression under certain conditions. In fact, obese young patients showing increased ACE2 expression in lung epithelial cells are typically characterized by the increased severity of COVID-19 [161,162]. On the other hand, relative to the upper airway epithelial cells, the human olfactory epithelium shows higher levels of the expressed ACE2 protein, suggesting that the initial site of SARS-CoV-2 infection is the upper, rather than the lower, airway [163]. These important findings provide an explanation for the COVID-19-associated olfactory dysfunction, such as common disturbances in the sense of smell, which were reported in 85% COVID-19 patients who participated in a large, multicenter European survey [164]. Furthermore, the lower prevalence of COVID-19 children can be explained (at least in part) by the lower levels of *ACE2* expression in the nasal epithelium of children relative to adults [165].

To address the role of SARS-CoV-2 tropism in the efficiency of COVID-19 transmission, Sungnak et al. looked at the single-cell transcriptome expression data in scRNA-seq datasets from different tissues, such as the respiratory tree, ileum, colon, liver, placenta/decidua, kidney, testis, pancreas, and prostate gland of healthy donors [166]. This analysis revealed that TMPRSS2, the primary protease important for SARS-CoV-2 entry, is highly expressed in different tissues, whereas the SARS-CoV-2 entry receptor *ACE2* is characterized by relatively low expression levels in all the tissues analyzed [166]. These findings indicated that at the initial stage of infection, ACE2, and not TMPRSS2, represents a limiting factor for viral entry [166]. The authors also showed that *ACE2* is more highly expressed (and co-expressed with viral entry-associated protease *TMPRSS2*) in nasal epithelial cells, specifically in a goblet and ciliated cells. This important finding explains an apparent contradiction between the rapid spread of the SARS-CoV-2 and the dependency of this virus on alveolar epithelial cells as the primary point of entry and viral replication. The fact that the SARS-CoV-2 entry receptor ACE2 is more highly expressed and co-expressed with the viral entry-associated protease TMPRSS2 in nasal epithelial cells indicates that these cells can serve as loci of original SARS-CoV-2 infection and also act as possible reservoirs for virus dissemination within a given patient and from person to person [166]. It was also pointed that reported data describe the peculiarities of *ACE2* expression in various tissues of healthy donors and that the gene expression landscape in the nose and other tissues can be drastically changed in the course of viral infection [166].

Furthermore, since in addition to lung and airways, *ACE2* is expressed in the ileum, colon, and kidney [166], other modes of COVID-19 transmission, which involve intestine, kidney, testis, and other tissues, should be considered. Special attention should be paid to the intestines, which express the highest level of ACE2. Earlier studies demonstrated that diarrhea was present in up to 70% of patients infected with SARS-CoV [167]. Furthermore, a recent case report demonstrated the presence of SARS-CoV-2 in the feces of a COVID-19 patient with an initial diarrhea episode [168]. Similar findings have been reported in other studies, indicating that tests of feces and urine samples for the presence of SARS-CoV-2 are warranted [169].

Another important question is whether the ACE polymorphism can serve as one of the factors promoting the high efficiency of the COVID-19 spread? Besides serving as a CoV receptor, ACE2 plays an important role in the regulation of the renin-angiotensin-aldosterone system (RAAS), which includes a cascade of vasoactive peptides, which coordinates key processes in human physiology and maintains plasma sodium concentration, arterial blood pressure, and extracellular volume [170]. Angiotensin I is a physiologically inactive decapeptide derived from angiotensinogen by the action of renin. It serves as a precursor for an octapeptide angiotensin II, which is the main RAAS effector that acts as an agonist for both angiotensin II receptors type 1 and type 2 (AT_1_R and AT_2_R, respectively). Angiotensin II is generated from angiotensin I by the action of ACE1. Angiotensin II is converted, by ACE2, to the heptapeptide angiotensin-(1–7), which is a vasodilator. ACE2 also converts angiotensin I to the nonapeptide angiotensin-(1–9), which is further processed by ACE1 to generate angiotensin-(1–7) that serves as an antagonist for the AT_1_R receptors and an agonist for the MAS1 receptor (also known as proto-oncogene Mas). Therefore, in RAAS, ACE2 acts as an inhibitor by cleaving a single residue from angiotensin I to generate angiotensin-(1–9) and via degrading angiotensin II to the angiotensin-(1–7) [171]. Therefore, down-regulation or depletion of ACE2 results in the distortions of the angiotensin II levels, which are linked to an overwhelming number of chronic and acute diseases [170]. SARS-CoV-2 infection down-regulates ACE2 expression, leading to the subsequent elevation of the plasma angiotensin II levels, which, in turn, correlate with the total viral load and deterioration of lung tissues [75,172]. In fact, plasma of the COVID-19 patients was shown to contain significant levels of angiotensin II when compared with healthy individuals [173]. Importantly, in addition to ACE2, ACE1 may also be related to the efficient spread of COVID-19. In fact, it is known that circulating and tissue concentrations of ACE1 can be altered by a genetic deletion/insertion (D/I) polymorphism in intron 16 of the *ACE1* gene, with the D allele being associated with reduced expression of ACE2 [174]. Based on the analysis of the D-allele frequency of the *ACE1* gene in samples from 25 different European countries, Delanghe et al. concluded that 38% of the variability of the COVID-19 prevalence could be attributed to the relative frequency of the *ACE1* D-allele and that there is a significant correlation between COVID-19-associated mortality and the prevalence of the *ACE1* D-allele [174]. These data suggest that ACE1 D/I polymorphism may be regarded as a confounder in the spread of COVID-19 [174]. These observations are in agreement with the known role of ACE1 in pulmonary infections caused by coronaviruses [175]. Therefore, the *ACE1* D/I genotype may affect the clinical course of the infection. In contrast to this conclusion, the analysis of the *ACE2* genomic structure revealed that some allelic variants of this gene would potentially offer resistance against SARS-CoV-2 [176].

To address an issue of the ACE2 multifunctionality that not only serves as a SARS-CoV-2 receptor but also acts as a key RAAS component participating in the generation of a multitude of vasoactive peptides coordinating several physiological processes, we recently conducted a comprehensive bioinformatics analysis of the predisposition of major players related to the SARS-CoV-2-AAS axis to intrinsic disorder and showed that all these proteins contain functional intrinsically disordered regions [177]. These observations represent a unique protein intrinsic disorder-based view of the RAAS-SARS-CoV-2 interplay and indicate the importance of the consideration of the intrinsic disorder phenomenon [177].

An important feature of SARS-CoV-2 is the ability of this virus to be transmitted from human to household pets (specifically cats and dogs) [178,179,180,181,182,183,184,185,186,187], indicating that such susceptibility of domesticated animals to SARS-CoV-2 would increase the transmissibility of this virus and worsen the infection-related situation because these pets and other domestic animals are almost in constant contact with family members and especially with the children [188,189,190]. It is known that the ACE2 is expressed in most vertebrates, and not all ACE2 can be equally efficiently utilized by SARS-CoV-2 as the receptors. It was also pointed out that not all pets are equally susceptible to SARS-CoV-2, with chimpanzees and monkeys being the most sensitive to this infection, and with mice being shown to be the least susceptible to SARS-CoV-2 [178,191]. Although previous studies were focused on the structural part of the interactions between the SARS-CoV-2 spike protein and the ACE2 proteins from different organisms, a different approach was utilized in a recent study, where the intrinsic disorder predispositions ACE2 proteins from different species were compared [190]. Based on this comparative intrinsic disorder predisposition analysis of the ACE2 proteins from different organisms, it was concluded that despite the overall rather high similarity between the resulting disorder profiles, there is a noticeable difference between these proteins in the disorder predispositions of their N-terminal regions (residues 19–83) involved in the interaction with the SARS-CoV-2 S protein [190]. These observations suggested that the affinity of ACE2-protein S interaction could be, at least in part, determined by the local peculiarities of the intrinsic disorder distribution within the S protein-binding region of ACE2 [190]. These data also provide important indications that the analysis of the intrinsic disorder predisposition in ACE2 can help to predict which species could be infected with SARS-CoV-2 via the ACE2 binding rout and, therefore, could serve as an intermediate host in the transmission of this virus [190].

It was recently indicated that, at least in part, the COVID-19 success in transmission could be attributed to the intra-host genomic diversity and plasticity of SARS-CoV-2 and its ability to form low-frequency polymorphic quasispecies [192,193]. This may mean three things [194]: (i) The presence of such viral quasispecies characterized by some sequence diversity can be responsible for the differences in coping with innate host defenses, packaging, replication kinetics, translation efficiency, and response to the antiviral therapies. (ii) The genetic diversity of such viral quasispecies that entered the cytoplasm could be responsible for their genetic cooperation, resulting in an increase in viral replication efficiency. (iii) Under selection pressure, population fitness can be enhanced via the group cooperation among the viral quasispecies, with such group cooperation being frequently seen when the number of infecting viral particles between passages is high [195]. The structure and dynamics of quasispecies of replicating RNA enable virus populations to persist in their hosts and cause disease. In fact, there is a critical interplay between the host and virus mutual influences (including, in some cases, the quasispecies organization), which represent the main driving force for the long-term survival of viruses in nature. The stability of virus particles may also play a relevant role in successful transmission [196]. The presence of quasispecies has previously been reported for SARS-CoV and MERS-CoV [193,197,198]. It is known that the substantial genetic diversity of RNA viruses is driven by recombination events [199,200]. In CoVs, such a high frequency of homologous recombination, which can reach the level of 25% through the entire CoV genome [201], can be attributed to the commonly observed discontinuous RNA synthesis [202]. Epidemic outbreaks caused by the pathogenic HCoVs, such as HCoV-OC43 [44], HCoV-NL63 [27], SARS-CoV [27,203,204], and MERS-CoV [205], are reported to be characterized by frequent genomic rearrangements of HCoVs. It should be mentioned that the S protein of SARS-CoV is the most divergent viral protein in all strains infecting humans [206,207]. The variations arise quickly in both C- and N-terminal domains of S protein, providing important means for the immunological escape [208]. Furthermore, the N-terminal region of S protein hosts a recombination hot-spot, indicating the genomic instability of SARS-CoV-2 over the poly-A and poly-U regions [192]. Often, the progress of infection is associated with virus adaptation to host environments. Variants of the same virus can differ in disease potential (virulence) [209,210].

**Figure 2:**
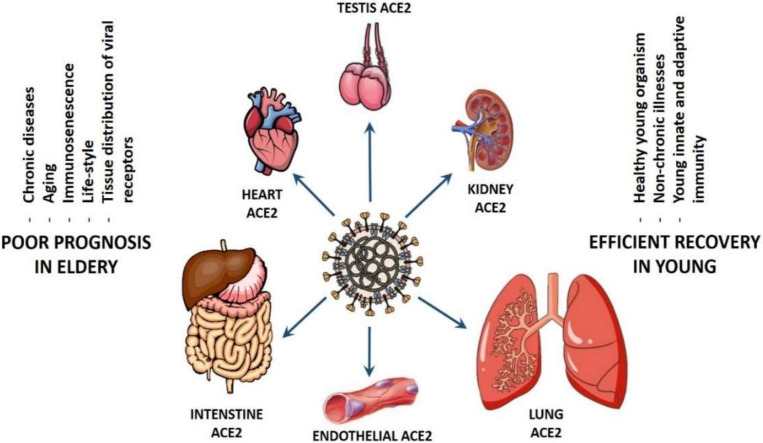
Suggested scenarios for the coronavirus disease 2019 (COVID-19) pathogenicity in old and young patients.

The COVID-19 tropism based on gender is a controversy. In fact, one study linked COVID-19 infection and transmission power to gender [211], whereas other researchers did not find any dependency of *ACE2* expression on gender on a single cell level [140], suggesting that the inter- and intra-gender viral transmission is equally efficient until this moment. However, the situation is completely different when comparing the patient susceptibility and the efficiency of COVID-19 transmission based on age (see Figure 2). It has been suggested that differential levels of ACE2 in the cardiac and pulmonary tissues of younger versus older adults maybe, at least, partially responsible for the spectrum of disease virulence observed among patients with COVID-19 [212]. Persons older than 60 years with chronic diseases, such as hypertension, diabetes, chronic obstructive pulmonary disease (COPD), as well as cardiovascular, cerebrovascular, liver, kidney, and gastrointestinal diseases, are more susceptible to the infection by SARS-CoV-2 and experience higher mortality when they develop COVID-19 [64,213,214]. In addition, patients older than 65 years generally have higher viral load lasting up to 14 days [215] in comparison to the younger patients, who have a much lower viral load that is undetectable within 1 week after onset [216]. 

The association between the viral load and the severity of COVID-19 has been reported [217]. Collectively, it seems the older people are more susceptible than younger people to COVID-19 hijacking, which may make them better hosts for virus passage. Generally, older persons, and especially those with chronic illness, are more susceptible to COVID-19. In fact, while many younger people experience no or mild symptoms of infection, older adults are highly susceptible to life-threatening respiratory and systemic conditions [218]. It seems that there are many factors defining why older people are more susceptible to COVID-19 and experience higher mortality when they develop COVID-19.

In fact, although aging is associated with many changes, one of the most pronounced transformations is the decline of the immune system, affecting both the innate and adaptive immune responses [219,220]. The process of chronological aging is known to affect various components of the immune response, leading to impaired host defense, defective vaccine responses, and a significantly higher risk of elderly persons developing life-threatening bacterial infections [219,221,222]. Aging affects all immune cells, including hematopoietic stem cells (HSCs), which maintain the immune system by producing all blood cells throughout the lifetime of an organism [223]. There are also age-related changes in the T cell compartment that are characterized by three main hallmarks: (i) Decrease in the number of naïve T cells related to the thymic involution [224,225]. (ii) Shrinking of the T-cell receptor (TCR) repertoire that determines antigenic diversity broadness and thus preconditions the successful elimination of pathogens from the system [226]. (iii) Increased proportion of the terminally differentiated oligoclonal effector memory T-cell population, especially those related to the control of persistent viral infections [227]. In old age, there is a decrease in the number and/or frequency as well as delay in the generation of the antigen-specific CD4 and CD8 T cell responses [228]. This generates a significant disturbance of a link between the early innate immune response and the recruitment of the antigen-specific T cells to the site of infection. Furthermore, the overall population of memory CD8 T cells is known to significantly change with age. Even though the total percentage of memory CD8 T cells is increased with age, the diversity of the repertoire of the naïve and memory CD8 T cell receptors is noticeably reduced in old age [229,230,231]. These changes in the immune system with age are allied with the poor immune responses of aged hosts to vaccines and viral infections [232,233,234].

Besides, in old humans, the number of peripheral B cells decreases, and the antigen-recognition repertoire of B cells and optimal pro-inflammatory cytokines production is altered [235]. As a consequence of the decreased generation of early progenitor B cells, the output of new naïve B cells is reduced [236,237], and, consequently, the longevity of the antigen-experienced memory B cells is increased [237]. Since class-switch recombination is impaired in memory B cells with aging [237,238], this may also contribute to the decline of the quality of humoral immune response [239]. The production of higher affinity protective antibodies in elderly individuals is impaired [240] due to the age-associated down-regulation of the activation-induced cytidine deaminase (AID), which is the enzyme for class switching, and its transcription factor E47 [241,242]. All these alterations can be related to the increased susceptibility of elderly people to infection with various pathogens [243,244].

Furthermore, as individuals age, they experience an increase in basal inflammation [245], which is now recognized as a global phenomenon known as inflammaging [246]. Inflammatory cytokines, including TNF and interleukin 6 (IL-6), are associated with increased risk for many diseases, including sarcopenia, osteoarthritis, and many infectious diseases [247,248,249]. The elderly are more susceptible to many infections, from those that are commonly diagnosed (influenza and pneumococcal pneumonia) [250,251] to those considered more exotic (such as anthrax and SARS) [248,252], due to their poor response to and control of infectious agents [253].

There are also some other age-related changes that can contribute to the increased susceptibility to infection. The NLRP3 (NACHT, LRR, and PYD domains-containing protein 3, where NACHT reflects a set of proteins containing this domain, e.g., (NLP family apoptosis inhibitor protein), CIITA (that is, C2TA or MHC class II transcription activator), HET-E (incompatibility locus protein from *Podospora anserina*) and TEP1 (that is, TP1 or telomerase-associated protein), whereas LRR and PYD stay for leucine-rich repeat and pyrin domain, respectively) inflammasome is a multiprotein complex consisting of the nucleotide-binding domain leucine-rich repeat-containing (NLR) family member NLRP3, the adaptor protein ASC (an apoptosis-associated speck-like protein containing a caspase recruitment domain (CARD) domain, also known as PYD and CARD domain-containing protein), and the cysteine protease caspase 1 [254]. The NLRP3 inflammasome can activate caspase 1 in response to cellular danger, resulting in the processing and secretion of proinflammatory cytokines—IL1β and IL18 [255,256,257]. Many studies reported high IL18 and IL1β levels in SARS, MERS, and COVID-19 patients, not only in the blood but also in lungs and lymphoid tissues, indicating the increased inflammasome activation. Maturation of IL1β (interleukin-1β) is achieved through the proteolytic cleavage of pro-IL1β by caspase 1, activation of which requires the formation of the NLRP3 inflammasome. When danger signals are sensed in the cells, NLRP3 is activated to recruit ASC and facilitate its oligomerization. For the full activation of the inflammasome, two signals are needed. The first of these signals stimulates the pro-IL1β transcription, whereas the second signal leads to the pro-IL1β cleavage [258].

A diverse array of stimuli can activate the NLRP3 inflammasome, including both pathogen-associated molecular patterns (PAMPs) and endogenous host-derived molecules indicative of cellular damage [259,260]. NLRP3 inflammasome responses are tightly regulated [261]. Using aged murine models of infection (influenza A virus (A/PR/8/1934(H1N1)), it was demonstrated that aged mice within 48 h post-secondary *Streptococcus pneumoniae* infection possessed increased morbidity and mortality. Increased susceptibility of aged mice was associated with decreased Toll-like receptors 1, 6, and 9 (TLR1, TLR6, and TLR9, respectively) mRNA expression and diminished IL1β mRNA expression. Examination of NLRP3 inflammasome expression illustrated decreased NLRP3 mRNA expression and decreased IL1β production in the aged lung in response to secondary *S. pneumoniae* infection [261]. Hoegen et al. used a pneumococcal meningitis model to demonstrate that the NLRP3 inflammasome could contribute to the increased host pathology instead of pathogen protection and clearance [262]. NLRP3 inflammasome is believed to be one of the major pathophysiologic components in the clinical course of patients with COVID-19 [263,264]. It has been shown that the NLRP3 inflammasome serves an important instrument in the development of acute lung injury (ALI) and acute respiratory distress syndrome (ARDS) [265]. It was also demonstrated that SARS-CoV viroporins (i.e., viral proteins with ion channel activity) E protein, ORF3a, and ORF8A act as ion-conductive pores in planar lipid bilayers and are required for maximal SARS-CoV replication and virulence [266]. Furthermore, there are data showing that these three proteins provoke the activation of the NLRP3 inflammasome [263]. For example, it was recently shown that the SARS-CoV ORF3a protein activates the NLRP3 inflammasome in lipopolysaccharide-primed macrophages by affecting K^+^ efflux and mitochondrial reactive oxygen species [267]. Another study showed that the SARS-CoV ORF3a accessory protein activates the NLRP3 inflammasome by promoting the TNF receptor associated factor 3 (TRAF3)-mediated ubiquitination of apoptosis-associated speck-like protein containing a caspase recruitment domain (ASC) [268]. Although the ORF8 protein of SARS-CoV-2 does not contain known functional domain or motifs, an aggregation motif VLVVL (residues 75–79) has been found in SARS-CoV ORF8B, which was shown to trigger intracellular stress pathways and activate the NLRP3 inflammasomes. However, this motif is apparently absent in ORF8 of the SARS-CoV-2 [264,269].

Apart from the cytokine storm observed in patients infected by the highly pathogenic HCoVs, other cell death programs, such as apoptosis and necrosis, might also contribute to the pathogenesis. Cell death is a double-edged sword that can play both antiviral and proviral roles during viral infection [270]. For example, ORF8a from the SARS-CoV was shown to trigger cellular apoptosis [271]. It was shown that the largest of the SARS-CoV accessory proteins, ORF3a, shares membrane insertion characteristics and channel functionality with necrotic effector molecules and interacts with receptor-interacting protein 3 (Rip3), which augments the oligomerization of ORF3a, causing causes necrotic cell death, lysosomal damage, and caspase-1 activation [272]. Apoptosis was detected in various HCoV-infected samples derived from not only the respiratory tract but also from the extrapulmonary sites [273]. Autopsy studies of SARS-CoV-infected tissues revealed the presence of apoptosis in the lung, spleen, and thyroid [274,275]. The apoptosis induced by SARS-CoV is caspase-dependent and could be inhibited by the Bcl2 overexpression or using the caspase inhibitors [276,277]. In 293 of ACE2 cells infected with SARS-CoV, several apoptosis-associated events were activated [278], namely cleavage of caspase-3, caspase-8, and poly(ADP-ribose) polymerase 1 (PARP), phosphorylation and inactivation of the eukaryotic translation initiation factor 2α (eIF2α), leading to the chromatin condensation, as well as activation of protein kinase R (PKR) and PKR-like endoplasmic reticulum kinase (PERK) [278]. Furthermore, HCoV-induced apoptosis was reported for several immune cells, such as macrophages, monocytes, T lymphocytes, and dendritic cells [279]. Infection of primary T lymphocytes by MERS-CoV induced DNA fragmentation and caspase 8 and 9 activation, indicating that, in this case, both extrinsic and intrinsic apoptotic pathways were activated [280]. Furthermore, MERS-CoV infection was shown to induce pyroptosis (which is a lytic and inflammatory mode of regulated cell death catalyzed by the caspase family) and over-activation of complement (which is an ancient molecular cascade that, being a part of the immune system, enhances the clearance potential of antibodies and phagocytic cells against microbes and damaged cells, as well as promotes inflammation and regulates attack at the membrane of pathogenic cells) in human macrophages [281].

The physical environment of the lung may also contribute to the efficiency of viral transmission. In fact, the elderly are more susceptible to many infections due to the aging-related changes in this environment [282,283], such as decreased strength of respiratory muscles, reduced lung elasticity, and lowered vital capacity [283]. As a result of all these changes, the expulsion of infectious agents through breathing, cough reflex, or sneezing is impaired. This is further complicated by the increased probability of the fluid and/or solid aspiration into the lungs, as well as age-associated inflammatory diseases, such as pulmonary fibrosis or chronic obstructive pulmonary disease (COPD) [284]. In fact, it was emphasized that both susceptibility to SARS-CoV-2 and severity of COVID-19 are systematically increased in the patients with COPD [285]. Alveolar epithelial cells (AETs) are responsible for the generation, secretion, and recycling of the lung mucosa or alveolar lining fluid (ALF), which is crucial for the correct lung maintenance [286]. Senescence of the AETs in the aged individuals is associated with the decreased lung recycling [286] that might lead to the inflammatory response in the lung tissue [287], which represents a part of the chronic low-grade inflammation that develops with advanced age and is known as systemic inflammaging [288]. These considerations imply that in old age, ALF might be characterized by an elevated inflammatory profile. In agreement with this hypothesis, significantly increased levels of TNF, IL-6, IL-1β, and other inflammatory cytokines were found in pulmonary fluids of aged humans [289]. Such increased inflammation within the lung mucosa is strongly connected to the specific changes in various innate molecular defense mechanisms. For example, ALF from elderly human subjects contained increased levels of the components of the complement system (e.g., complement C3β chain) and surfactant proteins A and D (SP-A, SP-D) [289].

Among different factors potentially affecting the susceptibility to SARS-CoV-2 and changing the outcomes and mortality amongst COVID-19 patients are smoking and vaping [285,290,291,292]. This is in line with the well-known general correlation between smoking and increased prevalence and mortality of infectious diseases [291], and with the fact that many COPD patients are smokers [285]. One should keep in mind, though, that existing data on the prevalence of smokers among COVID-19 patients and on the association between the COVID-19 outcomes and smoking are rather contradictory [290]. In fact, although some studies showed that smokers are more susceptible to COVID-19, and smoking is associated with more severe disease outcomes [293], several other studies pointed out the underrepresentation of active smokers among the COVID-19 patients [290] and indicated that active smoking is not associated with the COVID-19 severity [294]. Since these observations of smokers being protected from infection and severe complications of COVID-19 contradict the known association between morbidity and mortality of respiratory infections and cigarette smoking, the existence of a ‘smoker’s paradox’ in COVID-19 was proposed [290]. Among the possible molecular mechanisms of such protection are inhibition of SARS-CoV-2 entry into cells and replication caused by the smoking-induced increase in the nitric oxide levels in the respiratory tract, anti-inflammatory effects of nicotine, and reduced risk of a cytokine storm in COVID-19 associated with the dampened immune response in smokers [290]. However, systematic analysis of the existing literature pointed out that many results used in support of the smoker’s paradox-related claims are questionable and limited, indicating that extreme caution should be used while considering the protective effects of active smoking against COVID-19 [290].

As a continuation of the discussion of a link between smoking and COVID-19, it was shown that the lung and oral epithelial tissue samples of smokers are characterized by the up-regulation of ACE2 and TMPRSS2, which are the SARS-CoV-2 receptor and the transmembrane protease needed for the virus entry into host cells, respectively [295]. Importantly, this ACE2 and TMPRSS2 up-regulation was also associated with the up-regulation of the androgen pathway, suggesting that the smoking-mediated increased activity of the androgen signaling pathway itself and up-regulation of the central regulators of androgen pathways (e.g., HDAC6, CTNNB1, and SMARCA4) paired with the increased ACE2 and TMPRSS2 expression could represent a mechanism for the increased susceptibility of smokers to SARS-CoV-2 [295]. Importantly, the opportunity for SARS-CoV-2 infection of being androgen-mediated [296] via the androgen receptor-TMPRSS2 link, where the transcription of the TMPRSS2 is controlled by the androgen receptor activity [297], can represent a mechanistic explanation for the known sex-related differences in the COVID-19 vulnerability and lethality, with males typically being more susceptible to the infection [298,299,300]. This also suggests that androgen deprivation therapy, leading to the reduction of the TMPRSS2 expression, thereby limiting SARS-CoV-2 cellular entry, could potentially protect against severe complications from COVID-19 [301,302,303].

We conclude this overview of the pathogenic pathways and transmission potentials of HCoVs by considering an interplay between epigenetics and the coronavirus infection. This short section complements the description of molecular mechanisms regulating the pathogenesis of the emerging coronaviruses, which are complex processes that include virus–host interactions associated with the entry, egress, innate immune regulation, and control of various types of programmed cell death. Epigenetics studies how the genetic and non-genetic factors can regulate phenotypic variation. Typically, epigenetic effects are caused by external and environmental factors that alter host expression patterns and performance without any change in the underlying genotype. Therefore, epigenetic regulation links genotype and phenotype by promoting changes in the function of the gene locus without affecting the sequence of the underlying DNA. Some of the most common epigenetic modifications include chromatin remodeling, DNA methylation, histone modifications, and non-coding RNAs. These factors act as important regulators of the remodeling of host chromatin and alter host expression patterns and networks in a highly flexible manner. It was pointed out that viruses are able to regulate the host epigenome via a set of highly evolved, intricate, and well-coordinated processes, aiming at promotion of the robust virus replication and pathogenesis [304]. Some of these viral mechanisms to disturb and antagonize epigenetic regulatory programs of the host include interference with the histone modification enzymes of the host [305], interference with the chromatin remodeling machinery [306], and the presence of viral proteins that directly bind to the modified histones of the host [307,308]. For example, it was shown that the highly pathogenic H3N2 influenza A virus interferes with the epigenetic control of the gene expression to inhibit the initiation of the host innate immune response using histone mimicry (the C-terminal region of viral NS1 protein mimics the H3 histone tail and interacts with the transcription complex) [309,310]. SARS-CoV and MERS-CoV were shown to delay and/or antagonize pathogen recognition by successfully delaying interferon (IFN)-stimulated gene response [311]. This was achieved by modulation of the histone modifications (such as enrichment in H3K27me3 and depletion in H3K4me3) for a subset of genes, favoring a closed chromatin conformation that inhibits interferon-stimulated gene (ISG) expression [304,311]. In patients with systemic lupus erythematosus, who already have elevated ACE2 levels due to the hypomethylation and overexpression of *ACE2*, oxidative stress induced by SARS-CoV-2 infection resulted in exacerbation of these lupus-induced DNA methylation defects, leading to further *ACE2* hypomethylation accompanied by the overexpression of ACE2 and enhanced viremia [312].

## 4. Concluding Remarks

Data collected in this review clearly indicate that SARS-CoV-2 uses multiple ways for efficient transmission. It has a virion structure optimized for various environmental conditions, allowing this virus to use both respiratory and fecal-oral transmission modes. Its S protein has an amended structure for efficient interaction with the ACE2 receptor and is optimized for furin cleavage. Furthermore, S protein can be primed and activated by TMPRSS2, furin, and multiple non-furin proteases (e.g., plasmin). In addition to ACE2, SARS-CoV-2 can interact with other cellular peptidase receptors, such as ANPEP and DPP4, and also can utilize non-peptidase receptors, such as DC-SIGN1, CLEC4G, and CLEC4M. SARS-CoV-2 utilizes multiple ways for cellular entry (both non-endosomal and endosomal) and potentially uses various means of epigenetic control to inhibit the initiation of the host innate immune response. During the course of the pandemic, this CoV efficiently undergoes genomic rearrangements, thereby developing important means for the immunological escape. SARS-CoV-2 is engaged in intricate interplay with various host systems and pathways. It initiates cytokine storm and promotes various cell death programs, such as pyroptosis, apoptosis, and necrosis, which might contribute to the COVID-19 pathogenesis. This remarkably broad spectrum of means for the efficient SARS-CoV-2 transmission indicates that it is very unlikely that COVID-19 can be cured by targeting just one segment of this complex mosaic. A better understanding of various molecular mechanisms associated with all stages of SARS-CoV-2 infection is needed for finding the most appropriate approaches for COVID-19 prevention and treatment.
